# Immersive Virtual Reality Influences Physiologic Responses to Submaximal Exercise: A Randomized, Crossover Trial

**DOI:** 10.3389/fphys.2021.702266

**Published:** 2021-09-30

**Authors:** Sebastian Rutkowski, Patryk Szary, Jerzy Sacha, Richard Casaburi

**Affiliations:** ^1^Department of Physical Education and Physiotherapy, Opole University of Technology, Opole, Poland; ^2^Faculty of Physiotherapy, University School of Physical Education in Wroclaw, Wroclaw, Poland; ^3^Rehabilitation Clinical Trials Center, Division of Respiratory and Critical Care Physiology and Medicine, The Lundquist Institute for Biomedical Innovation at Harbor-UCLA Medical Center, Torrance, CA, United States

**Keywords:** virtual reality, VR, exercise capacity, exercise testing, immersion, heart rate variability

## Abstract

**Objectives:** This cross-sectional, randomly assigned study aimed to assess the influence of immersive virtual reality (VR) on exercise tolerance expressed as the duration of a submaximal exercise test (ET) on a cycle ergometer.

**Methods:** The study enrolled 70 healthy volunteers aged 22–25years. Each participant performed an ET with and without VR. Time- and frequency-domain heart rate variability (HRV) parameters were analyzed for the first 3min (T1), the last 3min (T2), and the time at which the shorter of the two tests terminated (Tiso). In the time domain, a SD of R–R intervals (SDNN) and a root mean square of successive R–R interval differences (RMSSD) in milliseconds were computed. The following spectral components were considered: low frequency (LF), high frequency (HF), total power (TP), and LF/HF ratio. The study was registered in ClinicalTrials.gov (NCT04197024).

**Results:** Compared to standard ET, tests in immersive VR lasted significantly longer (694 vs. 591s, *p*<0.00001) and were associated with lower HR response across the range of corresponding exercise levels, averaging 5–8 beats/min. In the multiple regression analysis, the ET duration was positively determined by male sex, immersion in VR, and negatively determined by HRT1 and RMSSDT1.

**Conclusion:** Exercising in VR is associated with lower HR which allowed subjects to exercise for a longer time before reaching target heart rate (HR). In addition, the increase in exercise duration was found to be related to an adjustment in autonomic nervous activity at a given work rate favoring parasympathetic predominance.

## Introduction

In everyday life, exercise tasks involving maximal exertion are seldom performed and, rather, a submaximal physical activity is preferred with lesser physiologic stress. Submaximal exercise tasks can be used to predict peak oxygen uptake (VO_2_peak), assess functional limitations, assess the ability to perform a rehabilitation program, or estimate the outcome of interventions (Loe et al., 2016). Cycle ergometers are the most common tools used in many European countries. Submaximal tests require the person to perform at less than their maximum capacity, and this procedure can be recommended for unfit people and for people with respiratory or cardiac risks (Bennett et al., 2016). Moreover, interventions are commonly sought that allow submaximal training to be performed in less stressful circumstances in order to achieve a better exercise tolerance. After an effective training program, high-intensity tasks may be performed with lower levels of pulmonary ventilation, oxygen uptake, and heart rate (HR; Casaburi et al., 1987a,b). However, stationary cycling is an exercise method unfamiliar to many people, and endurance is highly dependent on patient motivation. Therefore, the test may end prematurely, before reaching a cardiopulmonary endpoint. Throughout the test, the patient is unable to see the elapsed time, thus resulting in a monotonous pattern, which may lead to reduced patient attention and therefore reduced motivation to perform the task (Knecht and Kenning, 2016). A method of increasing patient motivation may be to perform exercises in a virtual reality (VR) environment (Tekerlek et al., 2017).

Virtual reality is based on creating a computer representation of objects, spaces, and events. It is a simulation of real situations *via* a high-quality user interface with real-time simulation and interaction through multiple sensory channels (Rutkowski et al., 2020). VR is created through specialized hardware and software, and the immersion is its key concept. The essence of immersion is its purposefulness and its ability to increase the effectiveness of therapy, in which the patient can become more involved. In order to achieve full immersion, the patient must see the full picture without any obstacles, and surrounding sound effects as well as body movements must be reproduced by an avatar in real time (Mazurek et al., 2019). Importantly, VR may work as a distractor from the stress of exercise (De Bruin et al., 2010) so that it can be more realistic and less boring (Ring, 1998). Presenting engaging scenarios may facilitate distracting the patient from negative symptoms (e.g., fatigue, dyspnea) during exercise (Rutkowski, 2021). However, before such technology might be prescribed to patients, it is important to determine whether the physiologic response would be similar to the traditional methodology. The analysis of the literature on the use of active virtual reality during exercise has shown a small number of papers describing the use of this type of apparatus (Huang et al., 2015; Gomez et al., 2018; Varela-Aldás et al., 2019; Mcclure and Schofield, 2020). Moreover, all previous research featured only the assessment of HR during workout sessions. Thus, we found a scarcity of literature evaluating how immersive VR training affects on HRV alterations during exercise test (ET) in healthy subjects. We therefore aimed to determine whether the implementation of immersive virtual reality during a submaximal exercise task would prolong the time until the target heart rate was achieved; we also investigated whether VR was capable of blunting the heart rate response and altering heart rate variability (HRV) associated with the exercise on a cycle ergometer in healthy individuals.

## Materials and Methods

### Participants

The study was carried out between December 2019 and January 2020 and enrolled 70 healthy young men and women volunteers aged 20–25years. Exclusion criteria included any diagnosed chronic diseases and any diseases and injuries of the locomotor system. None of the participants was taking any medications at the time of the study. None of the subjects was a regular cyclist, but all were familiar with riding a cycle ergometer. No participant had experience with the modalities used in the study. Participants were asked not to perform physical activity/exercise on the day of the study; in addition, participants rested approximately 30min before the experiment. Having considering the influence of the circadian rhythm on cardiovascular responses, the study was conducted from approximately 8a.m. to approximately 8p.m., took into account the entire day, and covered 4 consecutive days during the week due to the number of subjects. However, we took care to schedule all studies in a given participant at approximately the same time of day. Written informed consent was obtained from all participants, and the study was conducted in accordance with the Declaration of Helsinki. This study was approved by the Bioethical Commission of the Opole Chamber of Physicians (Resolution No. 289, 7 June 2019) and was registered in ClinicalTrials.gov (NCT04197024). The study was conducted at the Faculty of Physical Education and Physiotherapy of the Opole University of Technology. The data taken from human subjects were collected, managed, and protected by the research team. This study was designed as a single-blinded, crossover trial.

### Exercise Testing

**Figure 1:**
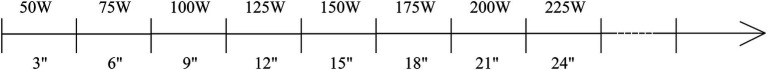
Test protocol.

The ET on an electronically braked cycle ergometer (Lode Excalibur Sport PFM) began with a unloaded cycling for 3min, then 50W for 3min followed by an incremental phase in which work rate increased by 25W every 3min (Zhang et al., 2017; Yamazaki et al., 2018; Figure 1). HR was continuously recorded using a Polar H10 monitor. These submaximal tests were terminated when the subjects reached 85% of the age-predicted maximal HR (calculated as 220 – age; Zhang et al., 2017). Participants were instructed during the test to maintain a pedaling frequency between 60 and 70 rotations per minute (rpm; Abrantes et al., 2012). In case of decrease/increase in the rpm, the tester provided verbal instructions.

### Virtual Reality Apparatus

**Figure 2:**
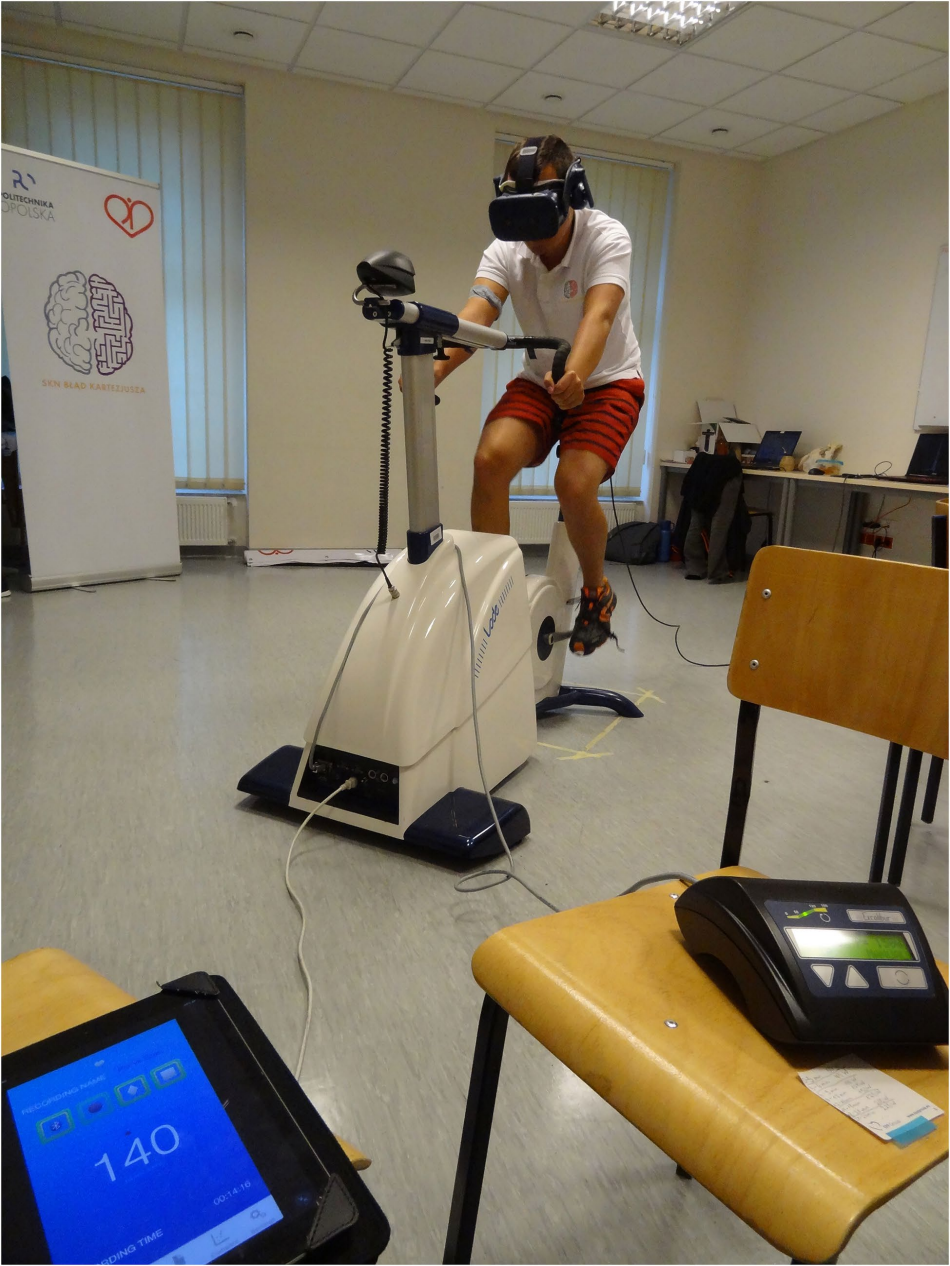
Research station.

The VR research station consisted of a HTC Vive Pro Goggle starter kit along with VR healthcare (aerobic exercise) VR cycling software (Thoth Technology Ltd., Ontario, Canada). An ankle mounted HTC Vive Tracker device was used to link the pedal speed with the VR images (Figure 2). Such a system enables its user to achieve a total immersion in the virtual world (Mazurek et al., 2019). The software contained several scenarios including Maya gardens, snowy pathways, or urban-style streets. In the virtual world, individuals saw bicycle contours and integrated counters indicating the speed, duration, and covered distance. The display of the VR scenario began with the first loaded pedaling phase.

### Heart Rate Variability

The Polar H10 monitor (Polar Electro Oy Inc., Kempele, Finland) was employed to record heart rate series at a sampling frequency of 1,000Hz. The device is recommended as the gold standard for RR interval assessments during intense activities to obtain HR and HRV (Gilgen-Ammann et al., 2019). All HRV analyses were carried out with the Kubios HRV Premium version 3.3.1 (HRV analysis, University of Eastern Finland). The R–R interval series were filtered with the artifact correction algorithm set in automatic mode (Tarvainen et al., 2014). In addition, visual inspection was performed to identify large R–R interval outliers (defined as 20% higher or lower than the preceding R–R interval) – in such cases, stronger artifact filtering was employed with suitable thresholds to eliminate the outliers. Time- and frequency-domain HRV parameters suitable for short-term recordings were obtained according to the Guidelines of Task Force of The European Society of Cardiology and The North American Society of Pacing and Electrophysiology (Camm et al., 1996). In the time domain, a SD of R–R intervals (SDNN) and a root mean square of successive R–R interval differences (RMSSD) in milliseconds were computed. Before calculating spectral HRV indices, a smoothness priors-based detrending approach was employed (smoothing parameter, Lambda value=500; Tarvainen et al., 2002), and then R–R interval series were interpolated to evenly sampled time signals with 4-Hz resampling rate. The detrended and interpolated R–R interval signals were used to calculate HRV spectra by using fast-Fourier transform (FFT) with Welch’s periodogram method (without overlap). The following spectral parameters were computed: low frequency (LF, 0.04–0.15Hz), high frequency (HF, 0.15–0.8Hz), and total power (TP, 0.04–0.8Hz). The wider frequency range for HF and TP was chosen because of the high respiratory rate seen during exercise and, consequently, a shift of the respiratory peak toward higher frequencies in the HRV spectrum (Lackner et al., 2020).

The time- and frequency-domain HRV parameters were calculated for every 3-min stage of the exercise test. The HRV indices derived from the first 3min of loaded exercise (50W, denoted as T1), the last 3min (the highest stage tolerated, denoted as T2), and 3-min stage at which the shorter of the two tests terminated (denoted as Tiso) of the exercises are presented.

### Study Design

This research was designed as a cross-sectional, randomly assigned study. All participants performed traditional ET and ET conducted in VR (ET+VR) in a random order. For each participant, the two tests were carried out on consecutive days.

### Statistical Analysis

As the exercise test incorporated with virtual reality has not been previously described in the literature, therefore, a series of the first 30 consecutive subjects undergoing both ET and ET+VR was used to estimate the sample size calculation. In that cohort, the average difference±SD in the duration of ET+VR and ET was 118±94s. Further, the average difference in SDNN (the most commonly recognized HRV parameters) at end exercise was −0.5±1.1ms; in order to power adequately for both outcomes, the latter was used for the sample size calculation. Assuming, 20% dropout rate and 5% problem with the HRV calculation due to artifacts, a sample size of 66 subjects was projected to be sufficient to show a significant difference in SDNN between ET+VR and ET, with an alpha value of 0.05 and a beta value of 0.1. To exclude the bias and validate all the results in this study (including the regression models), we perform all the statistical analyses in a resampling perspective according to the bootstrap procedure with 3,000 samples in order to give a more general value to the conclusions drawn (Davison and Hinkley, 1997). Continuous variables were presented as mean±SD or median and interquartile range (IQR), where it was appropriate according to the Kolmogorov–Smirnov normality test. Categorical variables were presented as numeric values and percentages. Differences between variables obtained during traditional ET and ET+VR were compared using Student’s paired *t*-test or the Wilcoxon signed-rank test. A stepwise linear regression analysis was performed to identify independent determinants of submaximal exercise test duration. Variables with skewed distribution (mostly HRV parameters) were logarithmically transformed to ensure normal distribution. Two models were built: the first model considered only parameters of the initial exercise stage (i.e., T1); the second model included potential determinants from the initial and the “isotime stage” at which the shorter of the two tests ended (i.e., T1 and Tiso). Factorial ANOVA was used to compare changes in HR with the incrementation of work rate in ET and ET+VR. The threshold probability of *p*<0.05 was taken as the level of statistical significance. All analyses were performed using NCSS 12 Statistical Software (2018). NCSS, LLC. Kaysville, Utah, United States, and STATISTICA 13 software (StatSoft, Cracow, Poland)^1^.

## Results

Seventy volunteers took part in this study; however, the analysis was performed on data coming from 65 participants because of recording errors in five participants in one of the two tests. Table 1 presents the physical characteristics of these 65 participants.

**Table 1:** Characteristics of participants completing the study (mean±SD).

Age, years	23.7±1.0
Male, *n* (%)	17 (26)
Body mass, kg	69.6±16.2
Body height, cm	171.1±10.3
BMI, kg/m^2^	23.7±3.7

*BMI, body mass index*.

**Figure 3:**
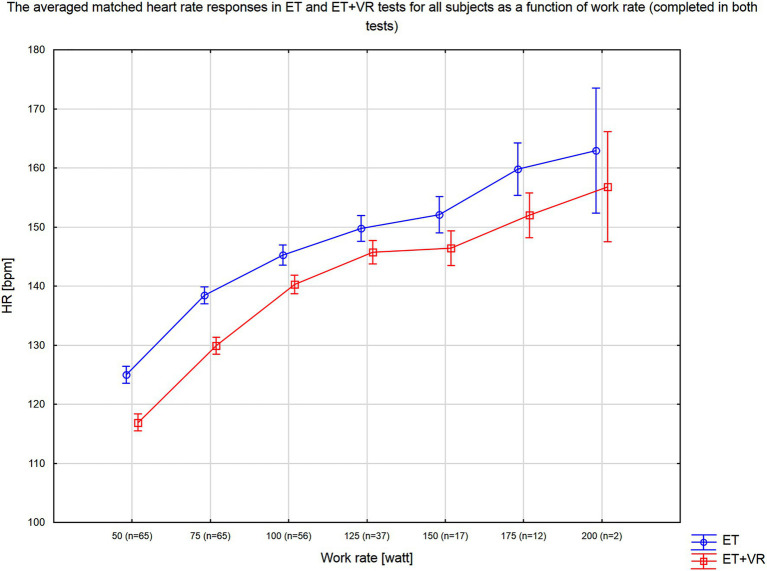
Average heart rate response as a function of work rate for tests conducted with (ET+VR) and without (ER) the virtual reality environment. N indicates the number of subjects who completed this work rate in both exercise tests.

In all tests, exercise was terminated when a subject reached the individualized heart rate target. Figure 3 presents the averaged matched heart rate responses in ET and ET+VR tests for all subjects as a function of work rate, which means the outcomes for the particular work rate that the participants completed in both tests. It can be seen that heart rate was consistently lower in the virtual reality tests over the full range of work rates. Since, in a given subject, exercise was stopped at the same heart rate in the two tests, the duration of the test and the peak work rate reflected the impact of VR on exercise tolerance. Of note, no order effect was present, i.e., the exercise duration and peak work rate did not differ between the first and second test. Importantly, the exercise time and peak work rate were higher in ET+VR than ET. Average exercise time was 103s (17.4%) longer with virtual reality (*p*<0.0000001; Table 2). Moreover, there were distinct differences in a number of HRV variables between the two modes of tests. In T1 and Tiso phases, the virtual reality was associated with lower HR and higher all absolute HRV parameters (i.e., SDNN, RMSSD, TP, LF, and HF; Table 2). Since HRV largely reflects the parasympathetic activity, the higher HRV and lower HR can be safely interpreted as a smaller withdrawal of the parasympathetic nervous system activity in T1 and Tiso. Conversely, in the T2 phase, HR was slightly higher (though not significantly), but SDNN, TP, LF, and HF (marginally) lower in VR, which was associated with longer exercise duration – suggesting that longer exercise required further reduction of the parasympathetic system activity (Table 2). The interpretation of the LF/HF (as well as nLF and nHF) changes is however much more uncertain, especially during exercises where the HF band must be extended beyond the standard limit, i.e., 0.4Hz (in our case, the upper HF limit was 0.8Hz). It was proven by Billman (2013) that the complex nature of LF power, its poor relationship to sympathetic nerve activation, and the nonlinear interactions between sympathetic and parasympathetic nerve activity make it impossible to delineate the physiologic basis for LF/HF with sufficient degree of certainty. According to Burr (2007) analysis of the normalized HRV indices, nLF and nHF are algebraically redundant, and both have simple monotonic nonlinear algebraic relationships with the LF/HF ratio. Therefore, all three indices reflect the same aspects of ANS balance and will be statistically equivalent in nonparametric statistical analyses based on rank-order properties of the dataset. Therefore, the interpretation of the LF/HF, likewise nLF and nHF changes can only be speculative, and the only reliable conclusions may be drawn from absolute HRV parameters.

**Table 2:** Responses to exercise with and without virtual reality (VR).

Characteristic	ET	ET+VR	*p* value
Exercise duration [s]	591.0 [406.0–738.5]	694.0 [580.0–880.0]	<0.01
Peak work rate [W]	123±34	142±32	<0.01
HR_T1_ [bpm]	125.7±13.5	118.3±12.6	<0.01
SDNN_T1_ [ms]	9.4 [6.0–15.6]	11.3 [8.0–15.5]	<0.01
RMSSD_T1_ [ms]	6.1 [3.4–9.5]	7.5 [5.1–11.4]	<0.01
TP_T1_ [ms^2^]	72.0 [23.8–179.5]	120.1 [46.2–225.4]	<0.01
LF_T1_ [ms^2^]	40.0 [17.2–105.0]	54.0 [28.4–135.3]	<0.05
HF_T1_ [ms^2^]	22.1 [7.9–51.2]	39.5 [14.1–92.7]	<0.01
LF/HF_T1_	2.7 [1.5–4.3]	2.0 [1.1–3.4]	<0.01
nLF_T1_	0.73 [0.6–0.81]	0.67 [0.52–0.77]	<0.01
nHF_T1_	0.27 [0.19–0.4]	0.33 [0.23–0.48]	<0.01
HR_Tiso_ [bpm]	157.7±3.3	147.4±8.3	<0.01
SDNN_Tiso_ [ms]	3.2 [2.6–3.9]	4.1 [3.1–5.6]	<0.01
RMSSD_Tiso_ [ms]	2.3 [1.9–2.8]	2.8 [2.2–3.8]	<0.01
TP_Tiso_ [ms^2^]	7.1 [4.9–12.1]	12.8 [7.4–27.9]	<0.01
LF_Tiso_ [ms^2^]	4.3 [2.1–8.5]	8.3 [4.2–18.2]	<0.01
HF_Tiso_ [ms^2^]	2.5 [1.4–4]	4.2 [2.4–8.7]	<0.01
LF/HF_Tiso_	1.9 [0.8–4]	1.9 [0.9–3.6]	0.80
nLF_Tiso_	0.66 [0.44–0.8]	0.66 [0.47–0.78]	0.80
nHF_Tiso_	0.34 [0.2–0.56]	0.34 [0.22–0.53]	0.80
HR_T2_ [bpm]	158.0±3.0	158.7±2.8	0.16
SDNN_T2_ [ms]	3.2 [2.6–3.9]	2.9 [2.3–3.6]	<0.01
RMSSD_T2_ [ms]	2.3 [1.9–2.8]	2.2 [1.8–2.8]	0.33
TP_T2_ [ms^2^]	7.0 [4.6–12.1]	6.0 [3.9–7.7]	<0.01
LF_T2_ [ms^2^]	4.2 [2.1–8.5]	3.0 [1.7–5.2]	<0.05
HF_T2_ [ms^2^]	2.5 [1.4–3.9]	2.3 [1.3–3.4]	0.08
LF/HF_T2_	1.9 [0.8–3.8]	1.5 [0.7–2.9]	<0.05
nLF_T2_	0.66 [0.44–0.79]	0.6 [0.41–0.74]	<0.05
nHF_T2_	0.34 [0.21–0.56]	0,4 [0.26–0.59]	<0.05

*ET, exercise test; VR, virtual reality; HR, heart rate; SDNN, SD of NN intervals; RMSSD, root mean square of successive differences; TP, total power; LF, low frequency; HF, high frequency; LF/HF, low frequency to high frequency ratio; Mean±SD; median [IQR];T1, the initial 3min loaded exercise period (50W); Tiso, the time at which the shorter of the two tests ended; T2, the last 3min loaded exercise period*.

Two multivariable regression analyses were performed to identify determinants of the exercise duration at which the target HR was achieved. In the first model, which considered only HR-related parameters taken at the initial exercise stage (50W), male sex, virtual reality, lower HR_T1_, and higher LF/HF_T1_ ratio were independently associated with a longer exercise duration (Table 3). In the second model, considering parameters taken from the initial and isotime stage of the exercise, male sex, virtual reality, lower HR_T1_, and lower RMSSD_T1_ were independently associated with a longer exercise duration (Table 4). A significant interaction (*p*<0.05) was detected between male sex and RMSSD_T1_, which probably explains the negative relationship of RMSSD_T1_ with the exercise duration, i.e., in fact, the males had lower RMSSD_T1_ but their exercise tests lasted longer. The statistical significance of all the results in this study was validated in the bootstrapping analysis.

**Table 3:** Predictors of submaximal exercise test duration in the model considering initial exercise stage (T1) variables.

Determinant	Multiple regression model for exercise time *R*^2^=0.75, *p*<0.0001	
	Standardized beta coefficient	*p* value
Male sex	0.47	<0.0001
Virtual reality test	0.16	<0.01
HR_T1_	−0.51	<0.0001
LF/HF_T1_	0.16	<0.001

*HR, heart rate; LF/HF, low frequency to high frequency ratio. Values at T1 are, for each subject, the average of that seen in the VR and non-VR test*.

**Table 4:** Predictors of submaximal exercise test duration in the model considering variables corresponding to the initial stage (T1) and isotime stage (Tiso).

Determinant	Multiple regression model for exercise time *R*^2^=0.76, *p*<0.00001	
	Standardized beta coefficient	*p* value
Male sex	0.5	<0.0001
Virtual reality test	0.13	<0.01
HR_T1_	−0.72	<0.0001
RMSSD_T1_	−0.28	<0.001

*HR, heart rate; RMSSD, root mean square of successive differences. Values at T1 are, for each subject, the average of that seen in the VR and non-VR test*.

## Discussion

The study shows that the immersive virtual reality stimulation leads to a reduced heart rate response during a submaximal exercise test, and consequently, to the subject reaching a higher work rate before the target heart rate was achieved. The differences between the tests with or without VR cannot be explained by a learning effect, since the order of tests was random and it was confirmed that the exercise duration and peak work rate did not differ between the first and second test. The changes in heart rate variability suggest that the alterations in the autonomic nervous system activity are responsible for the observed heart rate behavior in the virtual reality immersion. A reason for the lower heart rate (and, therefore, prolongation of the test time) in the VR environment seems likely to be based on a reduction in stress levels, as assessed by HRV analysis. In these healthy subjects, lower HR at the same work rate can denote better exercise tolerance since it may predict a lower exercise stress for a given work rate. A significant effect of gender on the duration of the tests was also noted, male gender correlated positively with test time length. These findings are in line with current evidence that, in healthy individuals, aerobic capacity is 15–30% lower in women than in men (Woo et al., 2006). This gender-dependent variation has been well recognized to be due to smaller heart size, lower hemoglobin levels, and less muscle mass in women (Belza and Warms, 2004). Furthermore, the difference in test duration may be related to intensity of LF component observed in males and females, as males tend to express sympathetic predominance, while females tend to manifest vagal predominance parasympathetic (Perini et al., 2000; Rosli et al., 2016).

Heart rate variability is commonly used as a noninvasive tool to monitor cardiac autonomic activity, and HRV responses to an exercise test may provide useful information about the autonomic reaction to the physical and psychological stress (Michael et al., 2017). In response to exercise, an increase in cardiac sympathetic neural activity (cSNA) and a decrease in cardiac parasympathetic neural activity (cPNA) are generally consistent with current understandings of cardiac autonomic regulation (White and Raven, 2014). Despite the limitations of HRV as an indirect measure of cardiac effect modulation, oscillations in cPNA (secondary to respiration) are thought to be a major contributor to beat-to-beat HR changes (Goldberger et al., 2006). Thus, the literature indicates that the analysis of the “cPNA-HRV measures” represents the determination of the parasympathetic component by the sinoatrial node (Hautala et al., 2003). Furthermore, measures of overall variability (e.g., SDNN and TP) are recognized as both parasympathetic and sympathetic cardiac activity, although these measures tend to be related to cPNA-HRV measures (Martinmaki et al., 2008). The response of the cPNA-HRV during higher intensity exercise is associated with a lower RMSSD, and this follows a relatively consistent curvilinear decay profile as a function of exercise intensity (Leicht et al., 2008). Previous reports indicate that HRV responses to stressors may predict aerobic exercise performance (Boullosa et al., 2012; D’Agosto et al., 2014) and may be altered in some chronic diseases (Treiber et al., 2003; Phillips, 2011). The literature indicates that exercise intensity is the main factor determining HRV response during physical activity, and most studies show a reduction of HRV in the initial phases of exercises (D'Agosto et al., 2014; Michael et al., 2017). According to Michael et al. (2017), in healthy subjects, a reduction of RMSSD during exercise follows a relatively consistent curvilinear decay profile as a function of exercise intensity at a moderate intensity is achieved at HR of ~120–140bpm or 50–60% VO_2_max. Thereafter, RMSSD does not change substantially, although slight increases may be observed at higher exercise intensities (e.g., HR>180bpm; Michael et al., 2017). Caruso et al. (2015) evaluated the use of HRV measurements during resistance training in patients with coronary artery disease. After 8weeks, they noticed an increase in RMSSD and SD1 accompanied by a significant decrease in average HR, i.e., an increase in the parasympathetic nervous system activity (Caruso et al., 2015).

In our study, during the initial exercise phase, HR and LF/HF ratio were significantly lower, but SDNN, RMSSD, TP, LF, and HF were higher in ET+VR than in ET. These findings indicate that, in virtual reality, the participants demonstrated higher parasympathetic activity both at the lowest work rate and also at the highest equivalent work rate (Tiso). In the final phase of testing (T2) parasympathetic activity was lower, and therefore, the sympathetic activity was most likely higher in VR, and thus enabling to perform longer before reaching their target heart rate. A previous study found that low HRV is associated with impaired regulatory and homeostatic autonomic functions, which reduce the body’s ability to cope with internal and external stressors (Kim et al., 2018). These results are in line with the work by Varela-Aldás et al. (2019) who evaluated effects of immersive virtual reality on heart rate in a small study (*n*=4) of athletes exercising at a constant speed on a treadmill. When a calming environment was provided, heart rate was lower; when displaying an exciting and threatening environment, heart rate reached higher values (Varela-Aldás et al., 2019).

Jacobson (1993) described four types of virtual reality: immersive virtual reality, desktop virtual reality (i.e., low-cost home-made virtual reality game console) also called a non-immersive, augmented virtual reality (where computer-generated data merge into a real-world image), and simulation (mixed) virtual reality (a combination of real people and physical environments with virtual people and places, either controlled by humans or by artificial intelligence; Jacobson, 1993). The concept of immersion means immersing the body and mind in a computer-simulated reality, which can dominate the real world. As our study shows, such a virtual immersion can significantly impact autonomic activity and, thereby, change the responses to submaximal physical activity. The review by Adamovich et al. (2009) suggests that a virtual reality immersive training in rehabilitation stimulates brain activity, provides suitable visual feedback during training, and makes patients motivated and interested. An increasing number of studies indicates the feasibility and effectiveness of using head-mounted VR to deliver biofeedback. High levels of immersion and sense of presence make VR a promising tool for training and research purposes (Cummings and Bailenson, 2016; Blum et al., 2020). According to the study by Koelsch and Jancke (2015), which addressed changes in HRV while listening to the music, SDNN appears to be lower during the exciting than tranquilizing music. Of note, music is a part of the full immersion; another element is the image. Solca et al. (2018) observed an increase in HRV associated with the reduction of the pain sensation in patients with pain syndrome during an immersion session of virtual reality. Blum et al. (2019) investigated the calm image (scenes from nature) influence during VR breathing session. Patients showed more calmness and lower HR after the session and performed their activities more precisely (Blum et al., 2019).

Another practical feature of our study is the use of low-cost equipment. With the advancement of technology, it is now possible and affordable to record heartbeats using wrist-worn or chest strap wearable devices (Haghi et al., 2017). Due to the low cost and discreetness of these devices, a larger population can measure their heart activity continuously, both in the context of 24-h measurements or during training (Li et al., 2019). Photoplethysmography (PPG) used with wristbands or smartwatches can provide a measurement of HRV. These wrist-worn wearable devices, however, can produce inconsistent RR-intervals, caused mainly by motion and mechanical artifacts induced by external stimuli (Morelli et al., 2019), which may affect the HRV analysis and potentially yielding inaccurate results (Kim et al., 2007). Another group of devices to acquire data for HRV analysis is chest straps, mainly used in athletes. The advantage of the chest straps with Bluetooth Low Energy is that it already provides RR intervals, so RR data does not require additional processing. These innovations can provide an alternative to “gold standard” ECG (Schafer and Vagedes, 2013). The Polar chest strap (used in this study) has been shown to provide valid and reliable measurements of HRV parameters in children (Speer et al., 2020) and in adult populations (Giles et al., 2016). The Polar H10 was reported to yield a high correlation to a three-lead ECG Holter monitor, whereas, during high-intensity activity, the Polar outperformed the Holter device (Gilgen-Ammann et al., 2019). It has been shown that both PPG and heart rate sensors provide an acceptable agreement for the measurement of RMSSD when compared with ECG (Plews et al., 2017). Furthermore, Bolkovsky et al. (2012), with the use of PPG, compared mobile phone cameras with the results of ECG. The results were not statistically different, which suggests that this approach removes the necessity to acquire and fit the HR strap (Bolkhovsky et al., 2012). Due to the high correlation of low-cost device results compared to ECG, their use for HRV measurements for monitoring of professional athletes (Lellamo et al., 2006; Plews et al., 2013, 2014; Bellenger et al., 2016; Rave et al., 2020), monitoring of capacity to return-to-play (Lai et al., 2017), or in athletic training programs (Boullosa et al., 2013; Flatt and Esco, 2016) have been implemented. It appears that a major advantage of chest straps (such as the Polar H10) is that they are waterproof, and its long battery life means that data collection in non-laboratory conditions is not limited (Hinde et al., 2021). Another advantage of these modern sensors is the access to raw data, which makes data analysis through professional software such as KUBIOS very easy. They also do not require clinical experience in applying the sensors on the subject’s body, which is required when performing ECG. In addition, unlike ECG monitoring, no wires are required between the electrodes and the monitoring system itself, potentially resulting in more reliable results (Billeci et al., 2018). Thus, wireless technologies provide a suitable tool for monitoring cardiovascular and autonomic parameters during exercise, quantifying the autonomic response during such activity, without interference, and possible pitfalls due to the weight of the system and the use of wires (Billeci et al., 2019).

A limitation to our research was that we did not carry out expired gas analysis. The direct measurement of ventilation and gas exchange would have provided information regarding the effect of VR on metabolic and ventilatory responses to exercise. Further, as we studied submaximal responses, we did not assess the effect of VR on aerobic exercise capacity. Another limitation of our study is the lack of blood lactate assessment. Blood lactate concentration is often measured during clinical exercise testing (Goodwin et al., 2007), as well as during performance testing of athletes (Manzi et al., 2009). Our study did not determine the effect of different types of VR, likewise, we did not control the consumption of food and caffeinated beverages in the hours prior to the tests. Moreover, it should be noted that the use of frequency-domain indices of HRV to assess autonomic control during exercise is debatable. Some studies suggest that spectral analysis with the use of FFT or short-time Fourier transform (STFT) analysis are a reliable and reproducible technique during exercise (Amara and Wolfe, 1998; Pichon et al., 2004), although some suggest that it is not possible to estimate the precise values of the HF, LF, and total power at higher intensities (Perini and Veicsteinas, 2003). An analysis using the maximum entropy method (MEM) to explain changes in HF and LF based on short time series of RR intervals seems reasonable, as does not depend on stationary data (Shiraishi et al., 2018). Finally, the R–R intervals were not recorded at baseline. The recording of the R–R intervals at rest before the sessions is relevant since it is possible to verify if the baseline values are similar between the sessions. All of these issues should be addressed in further studies.

## Conclusion

In healthy young subjects performing identical bouts of submaximal incremental cycle ergometer exercise, the immersive virtual reality lowered heart rate response at identical exercise levels which, in turn, allowed subjects to exercise to higher work rates before the target heart rate was achieved. Heart rate variability analysis revealed that this may be related to the functional alterations in the autonomic nervous system. Future studies are needed to investigate the long-term impact of VR on the exercise tolerance and the autonomic nervous system activity.

## Data Availability Statement

The raw data supporting the conclusions of this article will be made available by the authors, without undue reservation.

## Ethics Statement

This study was approved by the Bioethical Commission of the Opole Chamber of Physicians (Resolution No. 289 7 June 2019) and was registered in ClinicalTrials.gov (NCT04197024).

## Author Contributions

SR, PS, JS, and RC meet criteria for authorship as recommended by the International Committee of Medical Journal Editors (ICMJE). SR contributed to study concept and design. SR and PS conducted the experiment. SR, JS, and RC analyzed data. All authors interpreted and critically revised the manuscript, read and approved the final manuscript, and agree to be accountable for all aspects of the work in ensuring that questions related to the accuracy or integrity of any part of the work are appropriately investigated and resolved.

## Funding

The APC was funded by Faculty of Physical Education and Physiotherapy of Opole University of Technology.

## Conflict of Interest

The authors declare that the research was conducted in the absence of any commercial or financial relationships that could be construed as a potential conflict of interest.

## Publisher’s Note

All claims expressed in this article are solely those of the authors and do not necessarily represent those of their affiliated organizations, or those of the publisher, the editors and the reviewers. Any product that may be evaluated in this article, or claim that may be made by its manufacturer, is not guaranteed or endorsed by the publisher.

## Footnotes

^1^ ncss.com/software/ncss
